# Voice handicap, vocal fatigue, and voice-related quality of life in patients with multiple sclerosis

**DOI:** 10.1590/2317-1782/e20230320en

**Published:** 2025-02-03

**Authors:** Raí dos Santos Santiago, Jaya Miranda Carvalho de Araújo, Márcia Helena Cassago Nascimento, Carolina Fiorin Anhoque, Alana Tagarro Neves, Gabriel Trevizani Depolli, Bruno Batitucci Castrillo, Paula Zago Melo Dias, Regina Eliza Albano Vanzo, Carla Carvalho Nascimento, Valerio Garrone Barauna, Lívia Carla de Melo Rodrigues

**Affiliations:** 1 Universidade Federal do Espírito Santo – UFES - Vitória (ES), Brasil.; 2 Universidade Federal de São Paulo – UNIFESP - São Paulo (SP), Brasil.; 3 Hospital Universitário Cassiano Antonio Moraes – HUCAM - Vitória (ES), Brasil.

**Keywords:** Multiple Sclerosis, Dysphonia, Fatigue, Quality of Life, Voice Handicap, Voice Disorder

## Abstract

**Purpose:**

to describe sociodemographic characteristics of individuals with multiple sclerosis and correlate and compare vocal fatigue, voice handicap, and voice-related quality of life of individuals with and without the disease.

**Methods:**

Cross-sectional, quantitative study with 52 volunteers with multiple sclerosis and 52 control volunteers, matched by sex, age, and education level. Sociodemographic and clinical data were collected through a questionnaire and medical record analysis. Participants responded to the reduced Voice Handicap Index (VHI-10), Vocal Fatigue Index (VFI), and Voice-Related Quality of Life (V-RQOL). Correlational and comparative analyses were performed, with a 5% significance level (p < 0.05).

**Results:**

There was a greater predominance of females diagnosed with multiple sclerosis, with a mean age of 40 years, who graduated from high school, and with a relapsing-remitting disease course. Voice handicap was positively correlated with vocal fatigue, and voice handicap and vocal fatigue were negatively correlated with voice-related quality of life in both groups. Participants with multiple sclerosis exceeded the VHI-10 and VFI cutoff scores and were below the V-RQOL cutoff score.

**Conclusion:**

There was a prevalence of the disease in young, educated females with relapsing-remitting disease. The greater the voice handicap and/or vocal fatigue, the lower the voice-related quality of life in both groups. However, people with multiple sclerosis self-reported greater voice handicap and vocal fatigue and poorer voice-related quality of life.

## INTRODUCTION

Multiple Sclerosis (MS) is a chronic, inflammatory, demyelinating, and neurodegenerative disease of the central nervous system (CNS). Its cause is heterogeneous and multifactorial, and it is immune mediated by genetic, infectious, and environmental interactions, triggering an abnormal immune response and consequently myelin injury and axonal damage^(1)^.

The prevalence of MS varies in different parts of the world, distributed globally in areas of low, medium, and high prevalence. It is estimated that 2.8 million people live with MS worldwide, which represents a ratio of 1:3,000 people in young individuals aged 20 to 50 years. The disease's mean prevalence in Brazil is 15 cases per 100,000 inhabitants – the most common neurological cause of disability in young adults, being twice as high in women than men^(2)^.

MS is diagnosed through multiple signs and symptoms since there is no specific marker or diagnostic test. Hence, it requires objective evidence of the dissemination of typical MS lesions in time and space, which may include the patient's clinical history. It is also important to consider sociodemographic variables, such as age, sex, and education level, and their possible influences on the diagnosis and understanding of the disease^(2,3)^.

It can present as relapsing-remitting MS (RRMS), characterized by relapses with a remission phase without progression of disability; secondary progressive MS (SPMS), characterized by relapses without clear remissions and worsening of disability; primary progressive MS (PPMS), which appears later and is characterized by a slow and constant progression of the disease since diagnosis; and progressive-relapsing (PRMS), a rare form with constant decline from the beginning, with or without recovery after the flare-ups, but the disease continues to progress without remissions^(4)^.

Damage caused by CNS lesions in individuals with MS leads to changes in different functional systems, including oral communication, speech, and voice^(5)^, occurring in 40% to 50% of cases. Specific speech changes often affect the phonatory and respiratory systems in neurodegenerative diseases with neuromotor impairment, manifesting as dysarthrophonia^(6)^. This highlights the importance of knowledge in vocal rehabilitation, which can influence the ideal time to start treatment in MS cases^(7)^. However, until this study was conducted, no research had been identified that detailed the vocal characteristics for each course of the disease.

The predominant vocal changes in people with MS include changes in vocal quality with breathiness, roughness, instability, and manifestations of vocal fatigue^(5,8,9)^. Studies show that vocal fatigue manifests more frequently than hoarseness^(8)^, has a significant impact on the patient's quality of life^(5,9)^, and may occur in cases of MS due to the primary and secondary mechanisms of the pathology^(10)^. However, a literature review demonstrated that respiratory issues and vocal changes received less attention in the studies it found than other aspects of speech^(3)^.

Studies that used the Voice-Related Quality of Life Survey (V-RQOL) and the Voice Handicap Index (VHI) to measure these aspects in individuals with MS concluded that the latter is greater and the former is lower in these patients than in vocally healthy individuals^(11-13)^. So far, no studies have been found that used the Vocal Fatigue Index (VFI) in individuals with MS.

This study aimed to describe the sociodemographic characteristics of individuals with MS and correlate and compare vocal fatigue, voice handicap, and voice-related quality of life in individuals with and without MS to help understand vocal changes and manifestations in cases of MS.

## METHODS

This cross-sectional quantitative study was approved by the Ethics and Research Committee of the institution of origin under number 4.744.048.

The study included 52 volunteers with MS from the neurology outpatient clinic of a university hospital and 52 control volunteers recruited by matching sex, age, and education level. The study included volunteers with a definitive diagnosis of MS according to the McDonald criteria^(3)^, aged 18 years or older, and who agreed to participate in the study by signing an informed consent form. It excluded occupational voice users, smokers, and individuals diagnosed with other associated neurological diseases.

Initially, a specialized and experienced professional in the area applied a questionnaire addressing aspects of the sociodemographic profile of both groups and the clinical profile of participants with MS, collecting data such as self-reported sex, age, and education level. For participants with MS, it obtained data from medical records, covering the progression of the disease, time elapsed since diagnosis, medication use, flare-ups at the time of collection (i.e., emergence of new neurological symptoms or significant worsening of pre-existing symptoms, persisting for at least 24 hours)^(14)^, in addition to the score on the Expanded Disability Status Scale (EDSS), which quantifies disabilities throughout the course of the disease. The EDSS scale assesses impairment in eight functional systems: pyramidal, cerebellar, brainstem, sensory, bowel, bladder, visual, and cerebral. The higher the score, the greater the neurological impairment^(15)^.

The following patient-reported outcome measures (PROM) were also applied: a) 10-item Reduced Voice Handicap Index (VHI-10)^(16)^; b) Vocal Fatigue Index (VFI)^(17)^; and c) Voice-Related Quality of Life Survey (V-RQOL)^(18)^.

The VHI-10 measures voice handicap through a single total score, calculated by simply summing the responses to its items. It ranges from 0 to 40, where 0 indicates “no voice handicap” and 40 indicates “maximum voice handicap”^(15)^. Its cutoff is 7.5^(19)^.

The VFI is a robust instrument to self-assess vocal fatigue and its impact. It has 17 items distributed across four factors – seven items are related to "Fatigue and vocal limitation", three to "Vocal Restriction", four to "Physical discomfort associated with voice", and three to "Recovery with Vocal Rest", represented by factors 1, 2, 3, and 4, respectively. The items are scored according to the frequency of occurrence of symptoms, from "never" (0 points) to "always" (4 points). The total VFI is calculated by simply summing the items (inverting factor 4, as recommended by the PROM’s Brazilian validation), with a cutoff of 11.5^(17)^.

The V-RQOL assesses the impact of dysphonia on the individual's voice-related quality of life, through 10 items distributed in three domains: socioemotional, physical, and global, measured by simply summing all items. The domains have values ranging from 0 to 100 – the closer to 0, the worse the voice-related quality of life, and the ​​closer to 100, the better the voice-related quality of life^(18)^. The cutoff is 91.25^(19)^.

The data were tabulated and analyzed using GraphPad Prism 8 software for necessary statistical treatments. The study applied the Shapiro-Wilk normality test and used Fisher's exact and Mann-Whitney tests to compare the case and control groups. It also used pattern recognition by the Random Forest machine learning method to verify associations between the variables, combining two approaches: unsupervised (URF) to evaluate possible groupings among the samples and supervised (RF) to evaluate the most relevant variables. The Spearman Correlation coefficient was used for the correlation analyses, whose magnitudes were considered weak from close to 0 to 0.3 or -0.3, moderate from 0.3 (or -0.3) to 0.7 (or -0.7), and strong from 0.7 (or -0.7) to 1 (or -1)^(20)^. All analyses were performed using MATLAB version 13, with a 5% significance level (p < 0.05).

## RESULTS

Table 1 presents the sociodemographic data of both groups, with no statistically significant difference in terms of sex, age, or education level.

**Table 1:** Sociodemographic data of participating subjects

		**MS (n = 52)**	**Control (n = 52)**	**p-value**
				
Sex	Male	17 (32.7%)	16 (30.8%)	> 0.99
	Female	35 (67.3%)	36 (69.2%)	
				
Age		39.5(± 12.7)	39.3 (± 12.4)	0.96
				
				
Education level	Middle school	8 (15.4%)	6 (11.5%)	0.72
	High school	26 (50%)	27 (51.9%)	
	Higher education	18 (34.6%)	19 (36.6%)	

Mann-Whitney test. Significant when < 0.05

**Caption:** MS: multiple sclerosis.

Table 2 presents the participants’ clinical data: course of the disease, age at diagnosis, EDSS, duration of disease, number of flare-ups, and medications.

**Table 2:** Clinical data of MS group participants (n = 52)

**Course of the disease**		
Relapsing-remitting	(%)	43 (82.7%)
Secondary progressive	(%)	9 (17.3%)
		
**EDSS**		
0 – 3	(n)	38
3.5 – 7	(n)	11
7.5 – 8	(n)	3
		
Age at diagnosis	Median (min-max)	29 years (12 – 58)
Length of the disease	Median (min-max)	7 years (1 – 24)
Number of flare-ups	Median (min-max)	4 (1 – 12)
**Medication**		
Dimethyl fumarate	(%)	13 (25.0%)
Natalizumab	(%)	14 (26.9%)
Fingolimod hydrochloride	(%)	5 (9.6%)
Interferon beta-1	(%)	3 (5.7%)
Others	(%)	10 (19.2%)
None	(%)	7 (13.6%)

**Caption:** EDSS: Expanded Disability Status Scale

**Figure 1:**
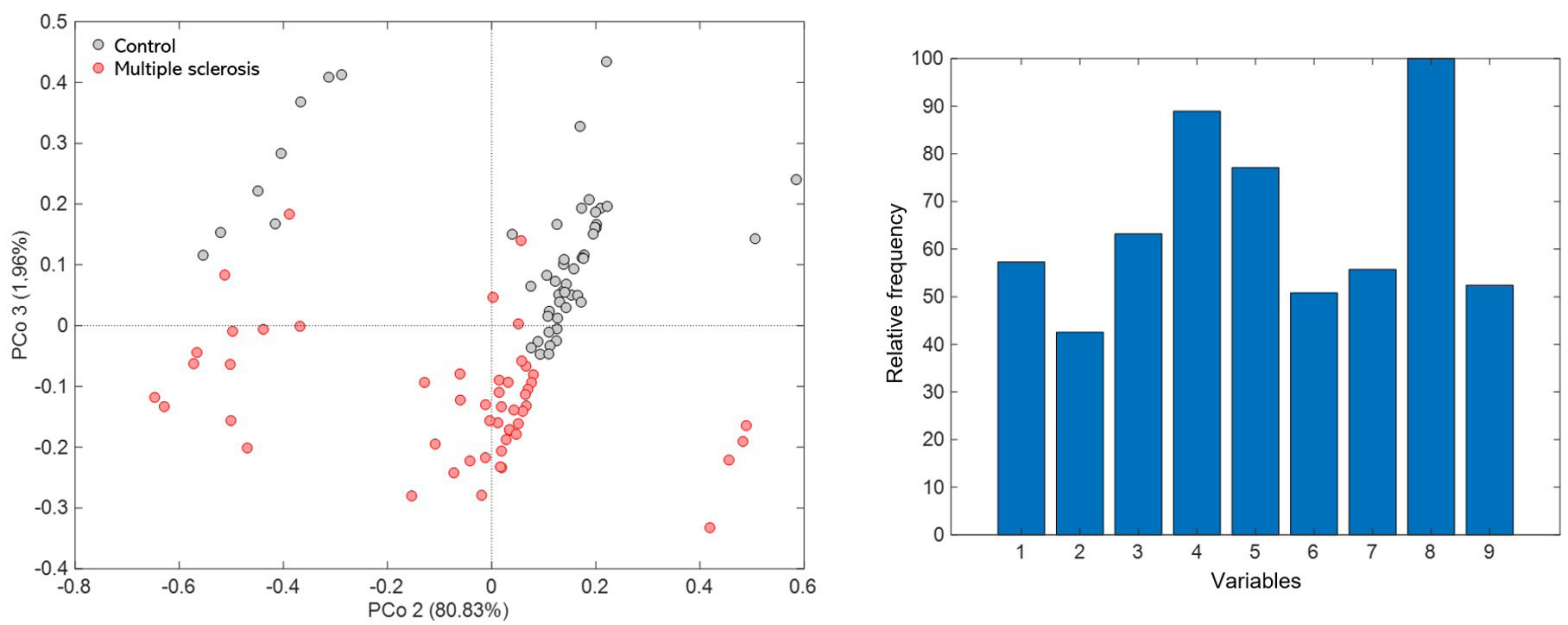
URF-PCoA model of the control (gray color) X multiple sclerosis groups (red color). Variables: 1) VHI; 2) VFI – fatigue and vocal limitation (factor 1); 3) VFI – Vocal restriction (factor 2); 4) VFI – Physical discomfort associated with voice (factor 3); 5) VFI – recovery with vocal rest (factor 4); 6) VFI – total score; 7) V-RQOL – total score; 8) V-RQOL – socioemotional score; 9) V-RQOL – physical score

Figure 1A presents the URF model for possible groupings and distinction of participants with and without MS. The variables analyzed were: 1) VHI; 2) VFI – factor 1; 3) VFI – factor 2; 4) VFI – factor 3; 5) VFI – factor 4; 6) VFI – total; 7) V-RQOL – total score; 8) V-RQOL – socioemotional score; 9) V-RQOL – physical - normalized (Min-Max). There was a tendency for separation between the groups, with a greater concentration of the control group in the upper quadrant (gray circles) and MS in the lower quadrant (red circles). Figure 1B presents the PCo3 loadings, indicating the most relevant and determining variables in the differentiation of the groups. In this case, the total V-RQOL score was the most significant and deterministic variable, followed by the VFI factor 3 score to distinguish the groups.

**Figure 2:**
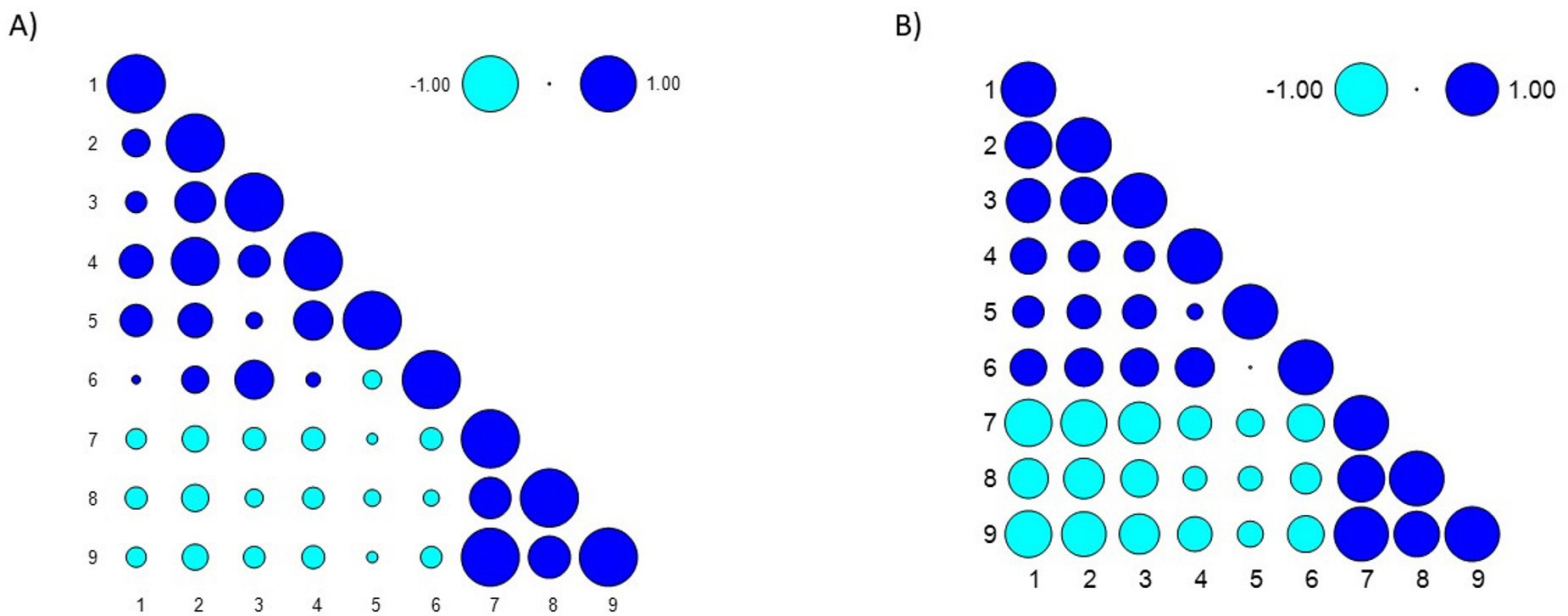
Correlation analysis of self-assessment protocols of control and MS subjects. (A) Control group; (B) MS group. Variables 1) VHI; 2) VFI – fatigue and vocal limitation (factor 1); 3) VFI – vocal restriction (factor 2); 4) VFI – physical discomfort associated with voice (factor 3); 5) VFI – recovery with vocal rest (factor 4); 6) VFI – total score; 7) V-RQOL – total score; 8) V-RQOL – socioemotional score; 9) V-RQOL – physical score

Then, the self-assessment protocols underwent correlation analyses. Figure 2A shows the control group, and Figure 2B shows the MS group.

In the control group, the VHI was moderately positively correlated with VFI factors 3 and 4 (p < 0.001; r 0.559; and p < 0.001; r 0.535, respectively). The VHI was also weakly negatively correlated with the V-RQOL total score (p 0.0198; r -0.3224), V-RQOL socioemotional score (p 0.0101; r -0.3535), and V-RQOL physical score (p 0.0220; r -0.3170).

In the MS group, the VHI was moderately to strongly positively correlated with all VFI factors (factor 1: p < 0.001; r 0.848; factor 2: p < 0.001; r 0.793; factor 3: p < 0.001; r 0.641; factor 4: p < 0.001; r 0.555; and total: p < 0.001; r 0.652). In contrast, the VHI was moderately to strongly negatively correlated with all V-RQOL scores (total: p < 0.001; r -0.855; socioemotional: p < 0.001; r -0.7166; and physical: p < 0.001; r -0.716). Furthermore, VFI factor 1 was moderately and strongly negatively correlated with all V-RQOL scores (total p < 0.001; r -0.8336; socioemotional p < 0.001; r -0.730; physical p < 0.001; r -0.810); factor 2 was so with all V-RQOL domains (total p < 0.001; r -0.750; socioemotional p < 0.001; r -0.664; physical p < 0.001; r -0.723); factor 3 was so with the V-RQOL total and physical domains (total p < 0.001; r -0.597; physical p < 0.001; r -0.618); and the total VFI score was correlated with the V-RQOL domains (total p < 0.001; r -0.662 socioemotional p < 0.001; r -0.544; physical p < 0.0001; r -0.6569).

The variables analyzed were similarly related in both groups, with correlation strength varying between them, although more strongly associated in the group with MS.

Table 3 presents a comparison analysis between the groups. The group with MS had higher scores in voice handicap and VFI factors 1, 2, 3, and 4 and lower scores in the domains of impact on voice-related quality of life than the control group.

**Table 3:** Comparison of self-assessment protocols of control and MS subjects

**PROM**	**Factor/Domain**	**Groups**	**Mean (SD)**	**Median**	**p-value**
**VHI-10**	Total	MS	8 (11)	3	**<0.001**
		CG	1.17 (2.50)	0	
**VFI**	Factor 1	MS	8 (9)	4.5	**<0.001**
		CG	1.51 (3.07)	0	
	Factor 2	MS	8 (4)	2	**<0.001**
		CG	0.28 (0.79)	0	
	Factor 3	MS	4 (5)	0.5	**<0.001**
		CG	0.76 (1.70)	0	
	Factor 4	MS	9 (5)	12	**<0.001**
		CG	3.07 (5.18)	0	
	Total	MS	18 (15)	12	0.0918
		CG	11.25 (4.97)	12	
**V-RQOL**	Physical	MS	80 (28)	95.8	**0.009**
		CG	97.49 (6.49)	100	
	Socioemotional	MS	86 (25)	100	**<0.001**
		CG	98.31 (6.03)	100	
	Total	MS	82 (25)	96.25	**<0.001**
		CG	97.22 (7.10)	100	

Mann-Whitney test. Significant when p < 0.05

**Caption:** PROM: patient-reported outcome measures; VHI-10: 10-item Voice Handicap Index; VFI: Vocal Fatigue Index; V-RQOL: Voice-Related Quality of Life; MS: group of volunteers diagnosed with multiple sclerosis; GC: control group.

## DISCUSSION

Voice and oral communication can serve as indicators of general health. In individuals diagnosed with MS, their assessment and monitoring can provide important information to determine the ideal time to initiate treatment^(7)^. However, studies have given less attention to vocal changes than other aspects of speech^(21)^. This study aimed to describe the sociodemographic characteristics of individuals with MS to deepen the understanding of vocal behavior in such cases. It also correlated and compared vocal fatigue, voice handicap, and voice-related quality of life between individuals with and without MS.

Individuals with MS had characteristics similar to those described in the literature, with a greater predominance of women, with a mean age of approximately 40 years, having finished high school, and diagnosed with RRMS^(22,23)^ (Tables 1 and 2). The characterization of participants’ clinical data can provide important information for understanding the prevalence of MS and its clinical condition^(2,3)^ – e.g., the level of education can be a positive point for understanding the disease and its treatment^(22)^. The lack of differences in sociodemographic variables between the groups (Table 1) was expected because it was a paired sample, thus strengthening the conceptualization of the control group.

The positive correlation between voice handicap and vocal fatigue demonstrates that the greater the self-reported voice handicap, the greater the self-reported vocal fatigue in both groups. Furthermore, the negative correlations between voice handicap and voice-related quality of life and between vocal fatigue and voice-related quality of life demonstrate that the greater the self-reported voice handicap and/or vocal fatigue, the lower the voice-related quality of life of people with and without MS (Figure 2). Studies have aimed to identify these symptoms and research rehabilitation methods. A relevant example is the study by Crispiatico et al.^(24)^, which used the Lee Silverman Voice Treatment (LSVT^®^) and recorded improvements in the vocal quality of all participants with MS after four weekly sessions over four weeks, especially individuals with moderate vocal fatigue. This suggests promising prospects for minimizing the vocal changes caused by MS.

Another factor that should be noted is that the relationship between these vocal aspects behaved similarly in both groups. This suggests that such aspects may be influenced by other factors, regardless of the presence of MS, and highlights the importance of addressing both voice handicap and vocal fatigue as significant components in the voice-related quality of life of individuals with and without MS.

However, individuals with MS often self-report handicaps^(5)^, vocal fatigue^(8)^, and poor voice-related quality of life^(12,13)^. This corroborates the data from the present study, given that the participants' overall means exceeded the VHI and VFI cutoff scores, and their V-RQOL mean score did not exceed the protocol cutoff – indicating poor voice-related quality of life. The same was not true for the control group (Table 3). Therefore, the VHI, VFI, and V-RQOL proved to be important tools in the self-identification of handicap, vocal fatigue, and voice-related quality of life of individuals diagnosed with MS. Exceeding the protocol cutoff score indicates high self-reporting of the vocal aspects analyzed. Thus, individuals with MS should be referred to a speech-language-hearing pathologist for multidimensional voice assessment and continuous monitoring.

Moreover, there were statistically significant differences in the VHI, VFI (except for its total), and V-RQOL between the groups (Table 3), corroborating data from the literature^(11)^. A control group study evaluated voice handicap and voice-related quality of life and performed auditory-perceptual evaluation in more than 60 individuals with MS, concluding that they have a greater voice handicap and that more than 40% of them had vocal changes in the grade of hoarseness, roughness, breathiness, and strain^(11)^. Although the present study did not investigate the auditory-perceptual evaluation of the participants' voices and that auditory-perceptual and self-assessment measures may not be related in people diagnosed with MS^(25)^, it is important to highlight that the VHI provides crucial information for the multidimensional vocal assessment, as evidenced by some studies, regardless of the auditory-perceptual evaluation^(11,26,27)^.

Considering that the group of participants with MS exceeded the protocols’ cutoff scores, it is recommended that speech-language-hearing pathologists include the PROMs used in this study to assess and monitor cases. Even in the absence of vocal complaints, these protocols can provide important information for the speech-language-hearing assessment and vocal self-perception comparison throughout the professional follow-up. They are important tools to assess the impact of the voice problem on quality of life^(28)^.

This study used new methods for the multivariate investigation of information. It used an RF model, whose robust, versatile, machine-learning approach is suitable for classification and regression tasks in supervised and unsupervised scenarios^(22)^. The URF model adopted in the study effectively distinguished the groups based on selected variables. The V-RQOL and the VFI voice-associated physical discomfort score (factor 3) were highlighted by the model employed, which demonstrates the relevance and applicability of these models in the analysis and distinction of groups based on the variables in question.

It is essential to highlight some limitations in our research, including the absence of vocal auditory-perceptual evaluation, considerations about aspects of the disease (such as time of diagnosis, number of flare-ups, and EDSS), and its management (medications used and possible side effects). Therefore, it is essential to conduct more targeted studies in this area to investigate in further detail the markers of dysphonia and their effects on the quality of life of patients with MS.

## CONCLUSION

This study found a prevalence of the disease in young, educated females diagnosed with RRMS. The relationship between voice handicap, vocal fatigue, and voice-related quality of life occurs similarly in individuals with and without MS. However, people with MS self-reported greater voice handicap and vocal fatigue and poorer voice-related quality of life.
