# Supplementary material for: Voice handicap, vocal fatigue, and voice-related quality of life in patients with multiple sclerosis
Source: Codas. 2025 Feb 3;37(1):e20230320. doi: 10.1590/2317-1782/e20230320en (PMC11801195; doi:10.1590/2317-1782/e20230320en)
Supplement: Translation

# Translation: Desvantagem, fadiga vocal e qualidade de vida em voz em pacientes com Esclerose Múltipla

**DOI:** 10.1590/2317-1782/e20230320pt

**Authors:** Raí dos Santos Santiago, Jaya Miranda Carvalho de Araújo, Márcia Helena Cassago Nascimento, Carolina Fiorin Anhoque, Alana Tagarro Neves, Gabriel Trevizani Depolli, Bruno Batitucci Castrillo, Paula Zago Melo Dias, Regina Eliza Albano Vanzo, Carla Carvalho Nascimento, Valerio Garrone Barauna, Lívia Carla de Melo Rodrigues

**Affiliations:** 1 Universidade Federal do Espírito Santo – UFES - Vitória (ES), Brasil.; 2 Universidade Federal de São Paulo – UNIFESP - São Paulo (SP), Brasil.; 3 Hospital Universitário Cassiano Antonio Moraes – HUCAM - Vitória (ES), Brasil.

**Keywords:** Esclerose Múltipla, Disfonia, Fadiga, Qualidade de Vida, Desvantagem Vocal, Distúrbio de Voz

## Abstract

**Objetivo:**

descrever características sociodemográficas de indivíduos com esclerose múltipla, correlacionar e comparar a fadiga, desvantagem vocal e a qualidade de vida em voz de indivíduos com e sem a doença.

**Método:**

Estudo transversal, quantitativo e com 52 voluntários com esclerose múltipla e 52 voluntários-controle, pareados por sexo, idade e escolaridade. Dados sociodemográficos e clínicos foram coletados com questionário e análise de prontuários. Os participantes responderam ao Índice de Desvantagem Vocal reduzido (IDV-10), Índice de Fadiga Vocal (IFV) e Qualidade de Vida em Voz (QVV). Análises correlacionais e comparativas foram realizadas, com um nível de significância de 5% (p<0,05).

**Resultado:**

Maior predomínio de participantes diagnosticados com esclerose múltipla do sexo feminino, média de 40 anos, ensino médio completo e curso da doença do tipo remitente-recorrente. Houve correlação positiva entre a desvantagem e a fadiga vocal, e correlação negativa entre a desvantagem e fadiga vocal com a qualidade de vida em voz em ambos os grupos. Além disso, os participantes com esclerose múltipla ultrapassaram as notas de corte do IDV-10 e do IFV e ficaram abaixo da nota de corte do QVV.

**Conclusão:**

Houve prevalência da doença em indivíduos jovens do sexo feminino, escolarizados e do tipo remitente-recorrente. Quanto maior a desvantagem e/ou a fadiga vocal, menor é a qualidade de vida em voz em ambos os grupos. No entanto, pessoas com esclerose múltipla autorreferem maior desvantagem e fadiga vocal, além de menor qualidade de vida relacionada à voz.

## INTRODUÇÃO

A Esclerose Múltipla (EM) é uma doença crônica, inflamatória, desmielinizante e neurodegenerativa do Sistema Nervoso Central (SNC). É de causa heterogênea, multifatorial e imunomediada por interações genéticas, infecciosas e ambientais, desencadeando resposta imunológica anormal e consequentemente lesão mielínica e dano axonal^(1)^.

A prevalência da EM difere em várias partes do mundo, distribuída globalmente em áreas de baixa, média e alta prevalência. Estima-se que 2,8 milhões de pessoas vivem com EM no mundo, o que representa uma proporção de 1:3.000 pessoas, em indivíduos jovens com idade entre 20 e 50 anos. No Brasil, tem sido encontrada prevalência média da doença de 15 casos por 100.000 habitantes. Configura-se como a causa neurológica mais comum de deficiência em jovens adultos, sendo duas vezes maior em mulheres comparada aos homens^(2)^.

O diagnóstico da EM é realizado por meio de múltiplos sinais e sintomas, uma vez que não existe marcador ou teste diagnóstico específico. É necessário obter evidências objetivas da disseminação no tempo e no espaço das lesões típicas da EM, o que pode incluir o histórico clínico do paciente. Além disso, é importante considerar variáveis sociodemográficas, como a idade, gênero e o nível de escolaridade, e suas possíveis influências no diagnóstico e na compreensão da doença^(2,3)^.

A EM pode se apresentar na forma remitente-recorrente (EMRR), caracterizada por recidivas com fase de remissão em que não há progressão da incapacidade; progressiva secundária (EMPS), caracterizada por recaídas sem remissões claras e piora da incapacidade; progressiva primária (EMPP), caracterizada por uma evolução lenta e constante da doença desde o diagnóstico, aparecendo mais tardiamente; e progressiva-recorrente (EMPR), uma forma rara com declínio constante desde o início, podendo ou não haver recuperação após os surtos, mas a doença continua a progredir sem remissões^(4)^.

Os danos ocasionados por lesões no SNC em indivíduos com EM acarretam alterações de diferentes sistemas funcionais, incluindo a comunicação oral, fala e voz^(5)^, manifestando entre 40% e 50% dos casos. Em doenças neurodegenerativas com comprometimento neuromotor, as alterações específicas da fala frequentemente afetam os sistemas fonatório e respiratório, manifestando-se como disartrofonia^(6)^, Isso ressalta a importância do conhecimento em reabilitação vocal, o que pode influenciar o momento ideal para iniciar o tratamento em casos de EM^(7)^. No entanto, até a realização deste estudo, não foram identificadas pesquisas que detalhassem as características vocais para cada curso da doença.

As alterações vocais predominantes em pessoas com EM incluem alteração na qualidade vocal com soprosidade, aspereza, instabilidade, além de manifestação de fadiga vocal^(5,8,9)^. Estudos mostram que a fadiga vocal se manifesta mais frequentemente do que a rouquidão^(8)^, apresenta importante impacto na qualidade de vida do paciente^(5,9)^ e pode se apresentar nos casos de EM pelos mecanismos primários e secundários da patologia^(10)^. No entanto, numa revisão de literatura foi demonstrado que questões respiratórias e alterações vocais receberam menos atenção dos estudos encontrados em comparação aos demais aspectos de fala^(3)^.

Em estudos que utilizaram os protocolos Qualidade de Vida em Voz (QVV) e o Índice de Desvantagem Vocal (IDV) para mensuração da qualidade de vida relacionada à voz e da desvantagem vocal de indivíduos com EM, concluiu-se que a desvantagem vocal é maior e qualidade de vida relacionada à voz é menor em indivíduos com EM quando comparada a indivíduos vocalmente saudáveis^(11-13)^. Até então, não foram encontrados estudos que utilizaram o Índice de Fadiga Vocal (IFV) em indivíduos com EM.

Na tentativa de auxiliar a compreensão sobre alterações e manifestações vocais em casos de EM, o objetivo deste estudo é descrever características sociodemográficas de indivíduos com EM, correlacionar e comparar a fadiga, desvantagem vocal e a qualidade de vida em voz de indivíduos com e sem EM.

## MÉTODO

Trata-se de um estudo transversal, quantitativo, aprovado pelo Comitê de Ética e Pesquisa da instituição de origem, sob o número 4.744.048.

Participaram do estudo 52 voluntários com EM, do ambulatório de Neurologia de um Hospital Universitário e 52 voluntários-controle recrutados por pareamento de sexo, idade e escolaridade. Foram incluídos voluntários com diagnóstico definitivo de EM de acordo com os critérios de McDonald^(3)^, com idade igual ou superior a 18 anos e que concordaram em participar do estudo a partir da assinatura do Termo de Consentimento Livre e Esclarecido (TCLE). Foram excluídos do estudo profissionais da voz, fumantes e pessoas diagnosticadas com outras doenças neurológicas associadas.

Inicialmente, um profissional especializado e experiente na área aplicou um questionário, que abordou aspectos relacionados ao perfil sociodemográfico de ambos os grupos e ao perfil clínico do grupo de participantes com EM. Dados como autodeclaração de gênero, idade e nível educacional foram coletados. Para os participantes com EM, as informações foram obtidas de prontuário, abrangendo a evolução da doença, tempo decorrido desde o diagnóstico, uso de medicamentos, presença de surto no momento da coleta (caracterizado pelo surgimento de novos sintomas neurológicos ou agravamento significativo de sintomas pré-existentes, persistindo por pelo menos 24 horas)^(14)^, além da pontuação na *Expanded Disability Status Scale* (EDSS), que quantifica as incapacidades ao longo do curso da doença. A escala EDSS avalia o comprometimento em oito sistemas funcionais: piramidal, cerebelar, tronco cerebral, sensorial, intestinal e vesical, visual e cerebral. Quanto maior a pontuação, maior é o comprometimento neurológico^(15)^.

Além disso, foram aplicados os seguintes *Patient Reported Outcome Measures* (PROM); a) Índice de Desvantagem Vocal reduzido, composto por 10 itens (IDV-10)^(16)^; b) Índice de Fadiga Vocal (IFV)^(17)^; e c) Protocolo de Qualidade de Vida em Voz (QVV)^(18)^.

O IDV-10 possibilita a mensuração da desvantagem relacionada à voz por meio de um único escore total, que é calculado pela soma simples das respostas aos seus itens. Esse escore pode variar de 0 a 40, em que 0 indica “nenhuma desvantagem vocal” e 40 representa “desvantagem vocal máxima”^(15)^. O IDV-10 possui o valor de corte de 7,5^(19)^.

O IFV é um instrumento robusto e permite a autoavaliação da fadiga vocal e seu impacto. Consiste em 17 itens distribuídos em 4 fatores, em que sete são relacionados à "Fadiga e Limitação Vocal", três à "Restrição Vocal", quatro sobre o "Desconforto Físico Associado à Voz" e três relacionadas à "Recuperação com Repouso Vocal", representados pelos fatores 1, 2, 3 e 4, respectivamente. Os itens são pontuados de acordo com a frequência de ocorrência dos sintomas, entre "nunca" (zero pontos) a "sempre" (quatro pontos). O total do IFV é composto pela somatória simples dos itens, com inversão do fator 4, como preconiza a validação brasileira do PROM e possui valor de corte de 11,5^(17)^.

O QVV permite a avaliação do impacto da disfonia na qualidade de vida relacionada à voz do indivíduo, por meio de 10 itens distribuídos em três domínios: socioemocional, físico e global, mensurado pelo somatório simples de todos os itens. Os domínios apresentam valores que variam de zero a 100, em que quanto mais próximo de zero, pior é a qualidade de vida relacionada à voz, enquanto valores mais próximos de 100 indicam melhor qualidade de vida em voz^(18)^, sendo 91,25 seu valor de corte^(19)^.

Os dados foram tabulados e analisados através do *software* GraphPad Prism 8 para tratamentos estatísticos necessários. O teste de normalidade aplicado foi o Shapiro-Wilk. Para comparação entre grupo caso e grupo controle, foi utilizado teste Exato de *Fisher* e *Mann-Whitney*. Para verificar as associações entre as variáveis, utilizou-se o reconhecimento de padrões pelo método de aprendizagem de máquina *Random Forest,* combinando-se duas abordagens: não supervisionado (URF) de modo a avaliar possíveis agrupamentos entre as amostras e supervisionado (RF) para avaliação das variáveis de maior relevância. Para as análises de correlação, o coeficiente de Correlação de *Spearman*, em que as magnitudes foram consideradas fracas se próximas de zero a 0,3 ou -0,3, moderada se entre 0,3 (ou -0,3) e 0,7 (ou -0,7) ou forte se entre 0,7 (ou -0,7) e 1 (ou -1)^(20)^. Todas as análises foram realizadas através da versão MATLAB 13ª, em que o nível de significância adotado foi de 5% (p < 0,05).

## RESULTADOS

A Tabela 1 apresenta os dados sociodemográficos de ambos os grupos, em que não demonstra diferença estatisticamente significativa quanto ao gênero, idade e escolaridade.

**Tabela 1:** Dados sociodemográficos dos sujeitos participantes

		**EM (n=52)**	**Controle (n=52)**	**p-valor**
				
Gênero	Masculino	17 (32,7%)	16 (30,8%)	> 0,99
	Feminino	35 (67,3%)	36 (69,2%)	
				
Idade		39.5(± 12,7)	39,3 (± 12,4)	0,96
				
				
Escolaridade	Ensino Fundamental	8 (15,4%)	6 (11,5%)	0,72
	Ensino Médio	26 (50%)	27 (51,9%)	
	Ensino Superior	18 (34,6%)	19 (36,6%)	

Teste de Mann-Whitney.

* Significativo se <0,05

Legenda: EM: esclerose múltipla

A Tabela 2 apresenta os dados clínicos dos participantes: curso da doença, idade no momento do diagnóstico, EDSS, tempo de doença, número de surtos e medicações.

**Tabela 2:** Dados clínicos dos participantes do grupo EM (n=52)

**Curso da doença**		
Remitente-recorrente	(%)	43 (82,7%)
Secundariamente progressiva	(%)	9 (17,3%)
		
**EDSS**		
0 – 3	(n)	38
3,5 -7	(n)	11
7,5 – 8	(n)	3
		
Idade de diagnóstico	Mediana (min – máx)	29 anos (12 – 58)
Tempo de doença	Mediana (min – máx)	7 anos (1 – 24)
Quantidade de surtos	Mediana (min – máx)	4 (1 – 12)
**Medicação**		
Furamato de dimetila	(%)	13 (25,0%)
Natalizumabe	(%)	14 (26,9%)
Cloridrato de fingolimode	(%)	5 (9,6%)
Interferon beta 1	(%)	3 (5,7%)
Outros	(%)	10 (19,2%)
Nenhum	(%)	7 (13,6%)

**Legenda:** EDSS: *Expanded Disability Status Scale*

Na Figura 1A, é apresentado o modelo de URF para possíveis agrupamentos e distinção dos participantes com e sem EM. As variáveis analisadas são: 1) IDV; 2) IFV - fator 1; 3) IFV – fator 2; 4) IFV – fator 3; 5) IFV – fator 4; 6) IFV – total; 7) QVV – escore total; 8) QVV – escore socioemocional; 9) QVV – físico – normalizadas (Min-Max). Nota-se que foi observada uma tendência de separação entre os grupos, com maior concentração do grupo controle no quadrante superior (representado pelos círculos cinzas) e EM no quadrante inferior (representado pelos círculos vermelhos). Na Figura 1B, são apresentados os "*loadings*" do PCo3, os quais indicam as variáveis mais relevantes e determinantes na diferenciação dos grupos. Neste caso, o escore total do QVV se apresentou como a variável mais significativa e determinística, seguido pelo escore do fator 3 do IFV para distinção entre os grupos.

**Figura 1:** Modelo URF-PCoA de grupos Controle (cor cinza) x Esclerose Múltipla (cor vermelha). Variáveis: 1) IDV; 2) IFV - fadiga e limitação vocal (fator 1); 3) IFV – restrição vocal (fator 2); 4) IFV – desconforto físico associado à voz (fator 3); 5) IFV – recuperação com repouso vocal (fator 4); 6) IFV – escore total; 7) QVV – escore total; 8) QVV- escore socioemocional; 9) QVV – escore físico

Posteriormente, foram feitas análises de correlação dos protocolos de autoavaliação. Na Figura 2A, é apresentado o grupo controle, e a Figura 2B grupo com EM.

**Figura 2:** Análise de correlação dos protocolos de auto-avaliação dos sujeitos Controle e EM. (A) Grupo controle e (B) grupo com EM. Variáveis 1) IDV; 2) IFV - fadiga e limitação vocal (fator 1); 3) IFV – restrição vocal (fator 2); 4) IFV – desconforto físico associado à voz (fator 3); 5) IFV – recuperação com repouso vocal (fator 4); 6) IFV – escore total; 7) QVV – escore total; 8) QVV- escore socioemocional; 9) QVV – escore físico

No grupo controle, houve correlação positiva moderada entre o IDV e os fatores 3 e 4 do IFV (p<0,001; r 0,559; e p<0,001; r 0,535, respectivamente). Além disso, houve correlação negativa fraca entre o IDV com o escore total do QVV (p 0,0198; r -0,3224), escore socioemocional do QVV (p 0,0101; r -0,3535) e escore físico do QVV (p 0,0220; r -0,3170).

Já no grupo com EM, houve correlações positivas moderadas a fortes entre o IDV com todos os fatores do IFV (fator 1: p < 0,001; r 0,848; fator 2: p<0,001; r 0,793; fator 3: p<0,001; r 0,641; fator 4: p<0,001; r 0,555 e total: p<0,001; r 0,652). Em contrapartida, neste mesmo grupo, houve correlação negativa moderada e forte entre IDV e todos os escores do QVV (total: p<0,001; r -0,855; socioemocional: p< 0,001; r -0,7166; e físico: p<0,001; r -0,716). Além disso, houve correlações negativas moderadas e fortes entre o fator 1 do IFV com todos os escores do QVV (total p< 0,001; r -0,8336; socioemocional p<0,001; r -0,730; físico p<0,001; r -0,810), entre o fator 2 com todos os domínios do QVV (total p < 0,001; r -0,750; socioemocional p < 0,001; r -0,664); físico (p < 0,001; r -0,723), entre o fator 3 do IFV com os domínios total e físico do QVV (total (p < 0,001; r -0,597; e físico p < 0,001; r -0,618), além de correlação do escore total do IFV com os domínios do QVV (total p < 0,001; r -0,662 socioemocional p < 0,001; r -0,544; físico (p < 0,0001; r -0,6569).

As variáveis analisadas apresentam relações semelhantes em ambos os grupos, com força de correlação variando entre eles, entretanto, mais fortemente associadas no grupo com EM.

A Tabela 3 apresenta análise de comparação entre os grupos, em que se observa maior escore de desvantagem vocal e dos fatores 1, 2, 3 e 4 do IFV e menores escores referentes aos domínios de impacto na qualidade de vida em voz no grupo de indivíduos com EM em comparação ao grupo controle.

**Tabela 3:** Comparação dos protocolos de auto-avaliação dos sujeitos com EM e controle

**PROM**	**Fator/Domínio**	**Grupos**	**Média (DP)**	**Mediana**	**p-valor**
**IDV-10**	Total	EM	8 (11)	3	**<0,001**
		GC	1,17 (2,50)	0	
**IFV**	Fator 1	EM	8 (9)	4,5	**<0,001**
		GC	1,51 (3,07)	0	
	Fator 2	EM	8 (4)	2	**<0,001**
		GC	0,28 (0,79)	0	
	Fator 3	EM	4 (5)	0,5	**<0,001**
		GC	0,76 (1,70)	0	
	Fator 4	EM	9 (5)	12	**<0,001**
		GC	3,07 (5,18)	0	
	Total	EM	18 (15)	12	0,0918
		GC	11,25 (4,97)	12	
**QVV**	Físico	EM	80 (28)	95,8	**0,009**
		GC	97,49 (6,49)	100	
	Socioemocional	EM	86 (25)	100	**<0,001**
		GC	98,31 (6,03)	100	
	Total	EM	82 (25)	96,25	**<0,001**
		GC	97,22 (7,10)	100	

Teste de Mann-Whitney. Significativo se p<0,05

**Legenda:** PROM: *Patient Reported Outcome Measures*; IDV-10: Índice de Desvantagem Vocal composto por 10 itens; IFV: Índice de Fadiga Vocal; QVV: Qualidade de Vida em Voz; EM: grupo de voluntários diagnosticados com Esclerose Múltipla; GC: grupo-controle

## DISCUSSÃO

A voz e a comunicação oral podem servir como indicadores da saúde geral, e em pessoas diagnosticadas com EM, sua avaliação e monitorização podem fornecer informações importantes para determinar o momento ideal para iniciar o tratamento^(7)^. No entanto, as alterações vocais têm recebido menos atenção em estudos em comparação com outros aspectos da fala^(21)^. Com o intuito de aprofundar a compreensão do comportamento vocal em casos de EM, o presente estudo buscou descrever as características sociodemográficas de indivíduos com EM. Além disso, foram realizadas correlações e comparações entre a fadiga, a desvantagem vocal e a qualidade de vida vocal entre indivíduos com e sem EM.

Os indivíduos com EM apresentaram características semelhantes às descritas na literatura, com maior predomínio em mulheres, com média de idade de aproximadamente 40 anos, com ensino médio completo e diagnosticadas com EM do tipo recorrente-remitente^(22,23)^ (Tabelas 1 e 2). A caracterização dos dados clínicos dos participantes pode trazer informações importantes para a compreensão da prevalência da EM e para o entendimento clínico^(2,3)^, como o nível de escolaridade, que pode indicar um ponto positivo para a compreensão da doença e de seu tratamento^(22)^. A ausência de diferenças das variáveis sociodemográficas entre os grupos (Tabela 1) já era esperada por ser uma amostra emparelhada, fortalecendo assim a concepção do grupo-controle.

A correlação positiva entre a desvantagem e a fadiga vocal demonstra que quanto maior a autorreferência de desvantagem vocal, maior a autorreferência de fadiga vocal em ambos os grupos. Além disso, as correlações negativas entre a desvantagem vocal e a qualidade de vida em voz, e a fadiga vocal e a qualidade de vida em voz demonstram que quanto maior a autorreferência de desvantagem vocal e/ou de fadiga vocal, menor a qualidade de vida relacionada à voz de pessoas com e sem EM (Figura 2). Estudos têm se dedicado à identificação desses sintomas e à pesquisa de métodos de reabilitação. Um exemplo relevante é o estudo conduzido por Crispiatico et al.^(24)^, que utilizou o método *Lee Silverman Voice Treatment* (LSVT^®^) e registrou melhorias na qualidade vocal de todos os participantes com EM após quatro sessões semanais ao longo de quatro semanas, principalmente indivíduos com fadiga vocal moderada, o que sugere perspectivas promissoras para minimizar as alterações vocais ocasionadas pela EM.

Outro fator que deve ser observado é de que o comportamento da relação entre os aspectos vocais pesquisados se assemelhou em ambos os grupos. Este dado sugere que os aspectos vocais pesquisados podem ser influenciados por outros fatores, independentemente da presença de EM, e destaca a importância de abordar tanto a desvantagem quanto a fadiga vocal como componentes significativos na qualidade de vida relacionada à voz de indivíduos com e sem EM.

Entretanto, a desvantagem^(5)^, a fadiga vocal^(8)^ e a baixa qualidade de vida relacionada à voz^(12,13)^ são frequentemente autorrelatadas por pessoas diagnosticadas com EM, corroborando os dados do presente estudo, tendo em vista que as médias gerais dos participantes ultrapassaram os valores de corte do IDV e do IFV e que sua média do QVV não ultrapassou o valor de corte do protocolo, o que sinaliza baixa qualidade de vida em voz. O mesmo não aconteceu com o grupo controle (Tabela 3). Logo, o IDV, o IFV e o QVV demonstraram ser ferramentas importantes na autoidentificação de desvantagem, fadiga vocal e qualidade de vida relacionada à voz de indivíduos diagnosticados com EM. A ultrapassagem do valor de corte dos protocolos sinaliza a elevada autorreferência dos aspectos vocais analisados. Assim, propõe-se o encaminhamento para a avaliação vocal multidimensional e o monitoramento contínuo por um fonoaudiólogo nos indivíduos com EM.

Ademais, houve diferenças estatisticamente significantes no IDV, no IFV (exceto por seu total) e no QVV entre os grupos (Tabela 3), corroborando dados da literatura^(11)^. Um estudo de grupo-controle que avaliou desvantagem vocal, qualidade de vida em voz e realizou julgamento perceptivo-auditivo em mais de 60 indivíduos com EM, concluiu que pacientes com EM apresentam maior desvantagem vocal e que mais de 40% apresentaram alteração vocal no grau geral, rugosidade, soprosidade e tensão^(11)^. Embora o presente estudo não tenha investigado o julgamento perceptivo-auditivo das vozes dos participantes e que as medidas perceptivos-auditivas e de autoavaliação podem não estar relacionadas em pessoas diagnosticadas com EM^(25)^, é relevante destacar que o protocolo IDV oferece informações cruciais para a avaliação multidimensional vocal, como evidenciado por alguns estudos, independentemente do julgamento perceptivo-auditivo^(11,26,27)^.

Ainda sobre a transgressão do valor de corte dos protocolos pelo grupo de participantes diagnosticados com EM, recomenda-se aos fonoaudiólogos a aplicação dos PROM utilizados neste estudo como parte da avaliação fonoaudiológica e monitoramento dos casos. Mesmo na ausência de queixas vocais, a utilização dos referidos protocolos pode fornecer informações importantes para a avaliação fonoaudiológica bem como para a comparação da autopercepção vocal ao longo do acompanhamento profissional e são ferramentas importantes na avaliação do impacto do problema de voz na qualidade de vida^(28)^.

Neste estudo novos métodos foram abordados para investigação das informações de forma multivariada. Utilizou-se um modelo RF que emprega uma abordagem robusta e versátil em aprendizado de máquina, adequada para tarefas de classificação e regressão em cenários supervisionados e não supervisionados^(22)^. O modelo URF adotado no estudo mostrou eficácia na distinção entre grupos com base em variáveis selecionadas. O QVV e o escore de desconforto físico associado à voz (fator 3) do IFV foram destacados pelo modelo empregado, o que demonstra a pertinência e aplicabilidade desses modelos na análise e distinção dos grupos com base nas variáveis consideradas.

É fundamental ressaltar algumas limitações em nossa pesquisa, incluindo a ausência de julgamento perceptivo-auditivo vocal, considerações sobre aspectos da doença (como tempo de diagnóstico, número de surtos e EDSS) e seu gerenciamento (medicações utilizadas e possíveis efeitos colaterais). Portanto, é imprescindível realizar estudos mais direcionados nessa área, visando investigar de maneira mais detalhada os marcadores de disfonia e seus efeitos na qualidade de vida dos pacientes com EM.

## CONCLUSÃO

No presente estudo, houve prevalência da doença em indivíduos jovens do gênero feminino, escolarizadas e com diagnóstico de EM do tipo remitente-recorrente. A relação entre a desvantagem vocal, a fadiga vocal e a qualidade de vida relacionada à voz ocorrem de maneira semelhante em indivíduos com e sem EM. No entanto, pessoas com EM autorreferem maior desvantagem e fadiga vocal, além de menor qualidade de vida relacionada à voz.
